# Resolution of interaural time differences in the avian sound localization circuit—a modeling study

**DOI:** 10.3389/fncom.2014.00099

**Published:** 2014-08-26

**Authors:** Brian J. Fischer, Armin H. Seidl

**Affiliations:** ^1^Department of Mathematics, Seattle UniversitySeattle, WA, USA; ^2^Virginia Merrill Bloedel Hearing Research Center, Department of Otolaryngology - Head and Neck Surgery, University of WashingtonSeattle, WA, USA; ^3^Department of Neurology, University of WashingtonSeattle, WA, USA

**Keywords:** sound localization, interaural time differences, avian brainstem, nucleus laminaris, ITD resolution

## Abstract

Interaural time differences (ITDs) are a main cue for sound localization and sound segregation. A dominant model to study ITD detection is the sound localization circuitry in the avian auditory brainstem. Neurons in nucleus laminaris (NL) receive auditory information from both ears via the avian cochlear nucleus magnocellularis (NM) and compare the relative timing of these inputs. Timing of these inputs is crucial, as ITDs in the microsecond range must be discriminated and encoded. We modeled ITD sensitivity of single NL neurons based on previously published data and determined the minimum resolvable ITD for neurons in NL. The minimum resolvable ITD is too large to allow for discrimination by single NL neurons of naturally occurring ITDs for very low frequencies. For high frequency NL neurons (>1 kHz) our calculated ITD resolutions fall well within the natural range of ITDs and approach values of below 10 μs. We show that different parts of the ITD tuning function offer different resolution in ITD coding, suggesting that information derived from both parts may be used for downstream processing. A place code may be used for sound location at frequencies above 500 Hz, but our data suggest the slope of the ITD tuning curve ought to be used for ITD discrimination by single NL neurons at the lowest frequencies. Our results provide an important measure of the necessary temporal window of binaural inputs for future studies on the mechanisms and development of neuronal computation of temporally precise information in this important system. In particular, our data establish the temporal precision needed for conduction time regulation along NM axons.

## Introduction

Unlike the visual or somatosensory system, the auditory system cannot rely on a spatial representation of signals on its receptor surface. To localize a sound source, it computes microsecond arrival time differences of sound between the two ears. These interaural time differences, or ITDs, are also used for sound segregation, the suppression of unwanted noise (“cocktail part effect”) (Blauert, 1997; Yin, 2002; Konishi, 2003). The primary structure of the brain where ITDs are encoded is an array of coincident detector neurons receiving binaural excitatory inputs in the medial superior olive (MSO) in mammals (Stotler, 1953; Rose et al., 1966; Goldberg and Brown, 1969; Yin and Chan, 1990) and the nucleus laminaris (NL) in birds (Jhaveri and Morest, 1982; Young and Rubel, 1983; Carr and Konishi, 1990; Overholt et al., 1992; Köppl and Carr, 2008). For decades, the avian auditory system has been a favorite model to study the mechanisms of ITD processing. In particular the sound localization circuits of chickens and barn owls have received a lot of attention (e.g., Young and Rubel, 1983; Carr and Konishi, 1990; Overholt et al., 1992; Kuba et al., 2006, 2010; Sorensen and Rubel, 2006; Seidl et al., 2010; Wang and Rubel, 2012). These circuits are used to address the open questions of the mechanisms involved in the development of neural circuits for processing temporally precise information (Seidl et al., 2010; Yamada et al., 2013) and the neural code used for sound localization (Harper and McAlpine, 2004; Salomon et al., 2012).

**Figure 1:**
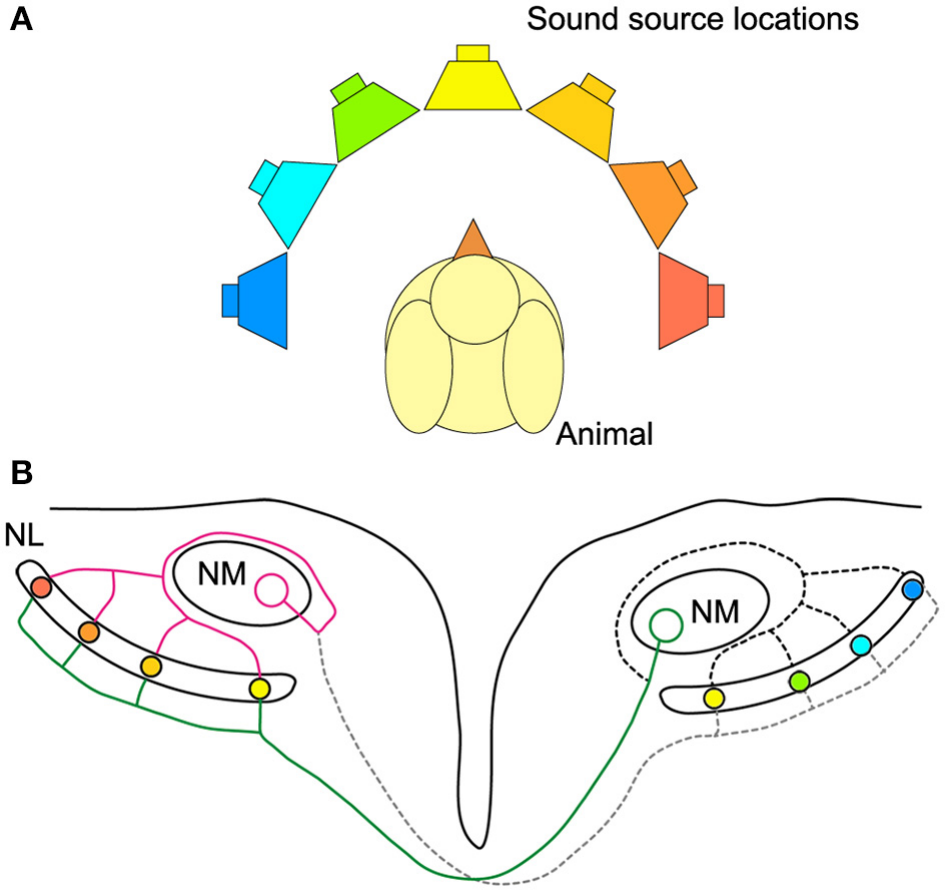
**Avian sound localization circuit. (A)** Color-coded speakers represent different sound source positions along azimuth. **(B)** Schematic representation of the sound localization circuit in the chicken auditory brainstem. NL cells are coincidence detectors and respond best to the sound source location with the corresponding color-coding. The ipsilateral axon terminals provide an isochronic input to the dorsal dendrites of NL, while the contralateral input is systematically delayed from medial to lateral. The ipsilateral and contralateral inputs to NL are provided by a single NM axon projecting to both NLs. Note that sound from straight ahead is encoded by neurons near the medial edge of NL and that most of the NL is tuned to sound source locations in the contralateral hemisphere. Magenta: ipsilateral axon branch, green: contralateral axon branch.

As sound arrives at the two ears, neurons in nucleus magnocellularis (NM) in the bird auditory brainstem receive phase-locked acoustically evoked input from the ipsilateral ear. NM neurons in turn project to neurons in NL on both sides of the brain (Figure 1). Interestingly, the signal from NM to NL is more temporally precise relative to sound phase than the auditory nerve (Fukui et al., 2006). This circuitry embodies a modified Jeffress model (Jeffress, 1948; Young and Rubel, 1983; Carr and Konishi, 1990; Overholt et al., 1992). In the Jeffress model, an axonal delay line compensates for external ITDs and enables coincident arrival of binaural inputs to neurons in NL (Figure 1B). Neurons in NL form a map of sound source locations in azimuth (Figure 1B). Only neurons receiving coincident binaural inputs respond maximally, and as such represent a specific sound source location. In other words, only a subset of neurons in NL is excited maximally by a particular ITD stimulus. An alternative to the place code for sound location is the two-channel code where neurons have best ITDs, or peak response, outside the natural range (the range of ITDs experienced naturally by the animal) and use the steepest part of the ITD curve to represent sound location (McAlpine et al., 2001; Harper and McAlpine, 2004). The code used for sound location in chickens remains in question, because it is unknown whether NL neurons can discriminate between ITDs within the natural range at all sound frequencies. In particular, the discrimination of ITD from NL neural responses has not been explored using a model that captures the diversity of responses observed in avian NL (Christianson and Peña, 2006; Köppl and Carr, 2008).

The binaural inputs to NL must be timed precisely, as the maximum ITD possible for chickens lies in the sub-millisecond range (Calford and Piddington, 1988; Hyson et al., 1994) (See Methods). Conduction velocity along NM axons providing the binaural input to NL neurons is regulated in a temporally precise manner to achieve coincident inputs (Seidl et al., 2010, 2014). Establishing and maintaining these coincident inputs provides a challenge during development when myelination occurs and as the head grows. The necessary precision of the inputs to NL provides an important constraint on mechanisms of the development of this circuit. The ITD discrimination limits for NL neurons place a bound on the required precision of inputs to NL.

We simulated ITD responses of single NL cells based on previously published data (Christianson and Peña, 2006; Köppl and Carr, 2008). Our results predict the minimum resolvable ITD when the maximum response of a NL cell is used to encode a particular ITD and when the slope of the ITD curve is used for discrimination. Our simulations indicate that the place code may be used for sound location at frequencies above 500 Hz, but that the slope of the ITD tuning curve must be used for ITD discrimination by single NL neurons at the lowest frequencies.

## Results

**Figure 2:**
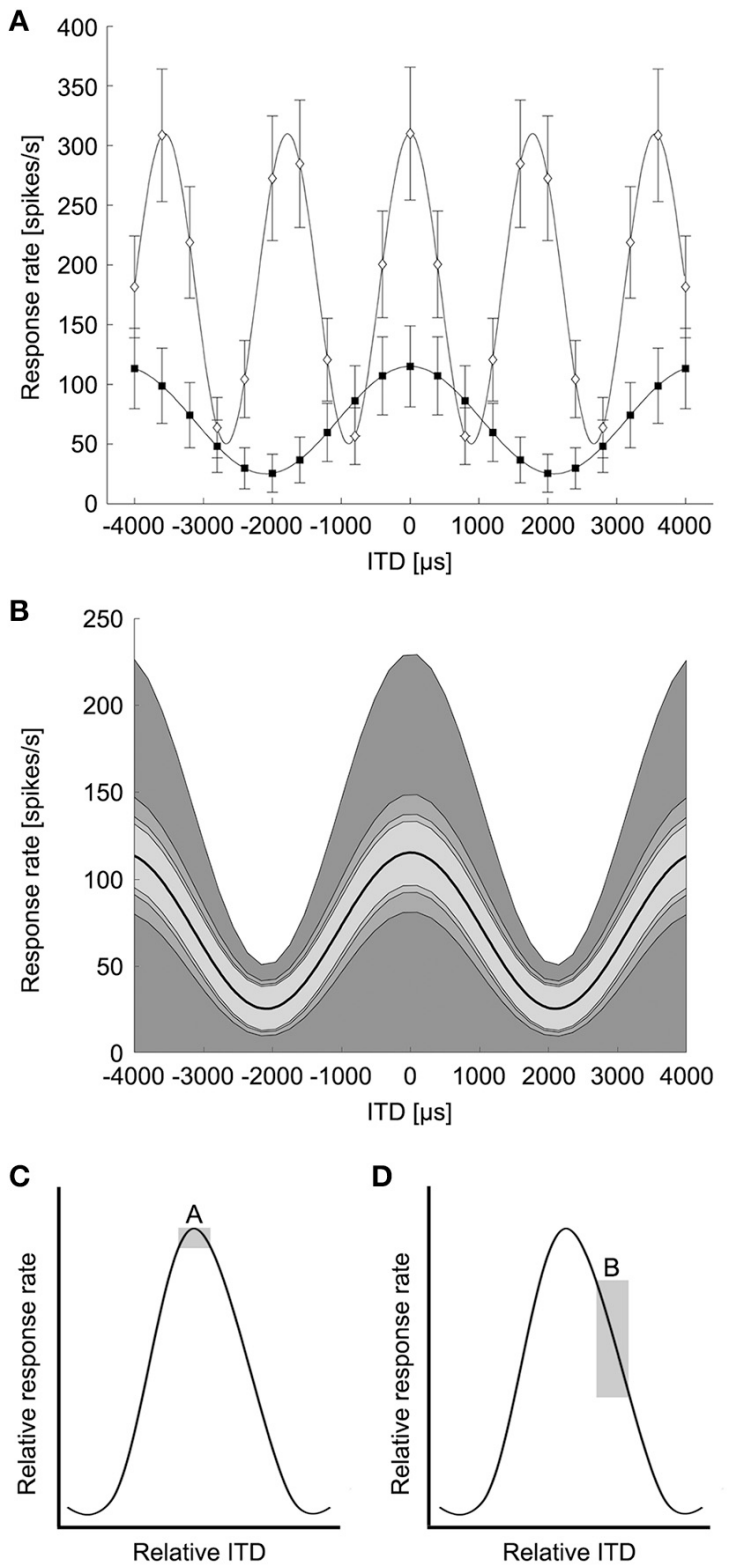
**Modeling of ITD tuning in avian NL neurons**. **(A)** Model neurons have sinusoidal ITD tuning curves with proportional Gaussian noise. A range of peak and trough firing rates were used at frequencies up to 4 kHz to model avian NL ITD tuning curves. White diamonds: 239 Hz, black squares: 562 Hz. Error bars are standard deviation (*k* = 2). **(B)** The variability of ITD responses was modeled as proportional Gaussian noise where the standard deviation of the spike count was given by the spike count raised to a power 1/*k*, *k* = 1, 2, 3, 4. The shaded regions indicate one standard deviation above and below the mean response (dark curve, 239 Hz). **(C)** Box A illustrates the possible difference in response rate (vertical dimension of box) if ITDs have to be discriminated around the peak of the ITD curve. **(D)** Box B illustrates the difference in response rate if the slope is used to discriminate between ITDs. (Adapted from Figure 6 in Hyson, 2005). The vertical dimension of box B is larger than box A, indicating a bigger change in response rate over the same ITD.

The classical concept of the Jeffress model predicts that different ITDs are encoded by particular cells responding maximally (Figure 1). The maximum excitation, or the peak of the ITD tuning curve, would determine the ITD a neuron encodes (Figure 2). Using the region of maximal slope might enable the system to resolve smaller ITDs (Joseph and Hyson, 1993; Hyson, 2005). We determined the minimum resolvable ITD in NL based on both the peak ITD and the point of steepest slope of the ITD tuning curve, modeled with pure tone stimuli.

### Modeling ITD tuning in NL

The ability to detect changes in ITD from the responses of coincidence detector responses in NL depends on the shape of the ITD tuning curve and the variability of the spiking responses (Rayleigh, 1907; Skottun et al., 2001; Butts and Goldman, 2006). ITD tuning curves of NL neurons are roughly sinusoidal, reflecting the underlying computation of cross-correlation between narrowband signals from the left and right ears (Stotler, 1953; Goldberg and Brown, 1969; Carr and Konishi, 1990; Yin and Chan, 1990; Fischer et al., 2008, 2011). While the sinusoidal pattern of ITD sensitivity is stereotypical, there is a diversity of responses in terms of trough firing rates and dynamic range (Christianson and Peña, 2006). NL neurons exhibit proportional noise where the variability of spiking responses increases with the mean firing rate (Reyes et al., 1996). There is also a diversity observed in the rate at which variability increases with mean firing rate. In the barn owl's NL, neurons have Fano factors ranging from approximately 0.25 to 1.5 (Christianson and Peña, 2006). Based on the published data on avian NL responses (Christianson and Peña, 2006; Köppl and Carr, 2008), we modeled ITD curves of avian NL neurons as a sinusoidal function of ITD plus a background firing rate:

$$r\left(ITD\right)=A\left[\text{cos}\left(2\pi f\left(ITD-ITD_{best}\right)\right)+1\right]+B$$

where *f* is the best frequency and *ITD_best_* is the best ITD of the neuron (Figure 2A). In the following we consider only the case where the neuron is stimulated at the best frequency. Note that the cosine tuning with a background response used here is motivated by avian NL responses (Christianson and Peña, 2006; Köppl and Carr, 2008) and differs from model responses used in previous studies of ITD coding (Harper and McAlpine, 2004). The ITD curve determines the spike count response of the neuron over a 100 ms stimulus presentation. There is no evidence for frequency-dependence in the parameters of the tuning curves. Therefore, at each frequency the background response *B* varied between 0 and 25 spikes/stimulus, in steps of 1 spikes/stimulus, and the amplitude *A* varied between 2 and 15 spikes/stimulus, in steps of 1 spikes/stimulus (Christianson and Peña, 2006; Köppl and Carr, 2008). The firing rate variability of NL neurons varies with the mean firing rate, but the variability can be lower than expected from a Poisson model (Christianson and Peña, 2006). The variability of ITD responses was modeled as proportional Gaussian noise where the standard deviation of the spike count σ(*ITD*) was given by the spike count raised to a power between ¼ and 1:

$$\sigma \left(ITD\right)=r{\left(ITD\right)}^{1/k}$$

where *k* = 1, 2, 3, or 4 in order to produce Fano factors above and below one as seen in NL (Figure 2B) (Christianson and Peña, 2006). We generated model neurons with combinations of model parameters *A*, *B*, and *k* covering the given ranges yielding 1456 total model neurons. This model produced the range of ITD responses observed in avian NL (Figure 2A, compare Figure 5A of Köppl and Carr, 2008).

### Minimum resolvable ITD

**Figure 3:**
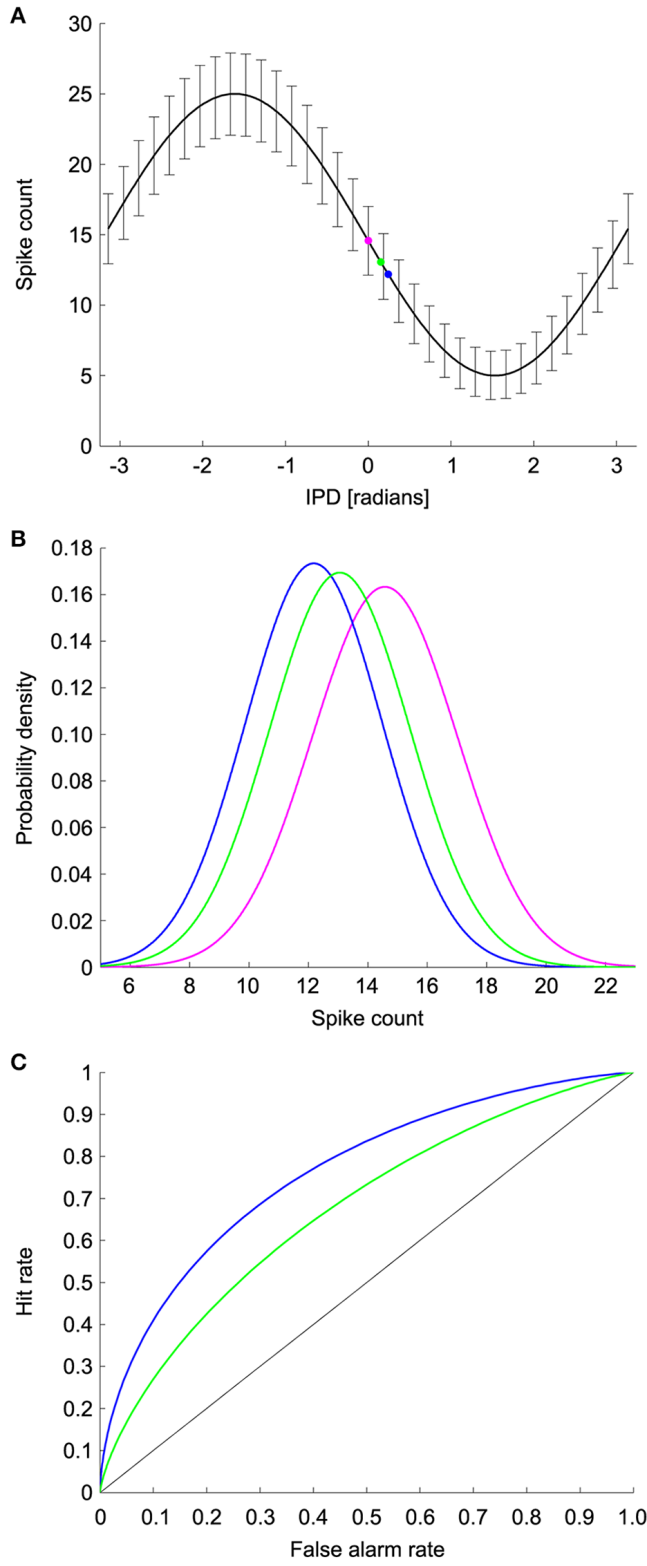
**ROC analysis. (A)** Model IPD tuning curve where the error bars are the standard deviation. The magenta dot corresponds to the spike count at a reference IPD. The blue and green dots correspond to the spike counts at two test IPDs. **(B)** The spike count distributions for the reference and test IPDs. Discrimination between the reference IPD (magenta) and green test IPD is more difficult than discrimination between the reference IPD and the blue test IPD due to the difference in overlap of the spike count distributions. **(C)** ROC curves for discrimination between the reference and test IPDs. The area under the ROC curve is the percent correct in the discrimination task.

We used ROC analysis to calculate the minimum resolvable ITD, denoted Δ*ITD*, from coincidence detector responses in NL (See Methods and Figure 3) (Bradley et al., 1987; Skottun, 1998; Skottun et al., 2001; Köppl and Carr, 2008). The ROC analysis uses the probability distributions of the spike counts at each ITD to find the smallest difference in ITD that can be discriminated with an accuracy of 75% correct. Responses were simulated for a 100 ms time window, which is consistent with behavioral integration time in owls (Knudsen et al., 1979). Given the frequency-independence in the model parameters, we first calculated the minimum resolvable interaural phase difference (IPD), denoted Δ*IPD*, using the model

$$r\left(IPD\right)=A\left[\text{cos}\left(IPD-IPD_{best}\right)+1\right]+B$$

where *IPD* = 2π*fITD*. The minimum resolvable ITD was then computed as

$$\Delta ITD=\frac{\Delta IPD}{2\pi f}.$$

**Figure 4:**
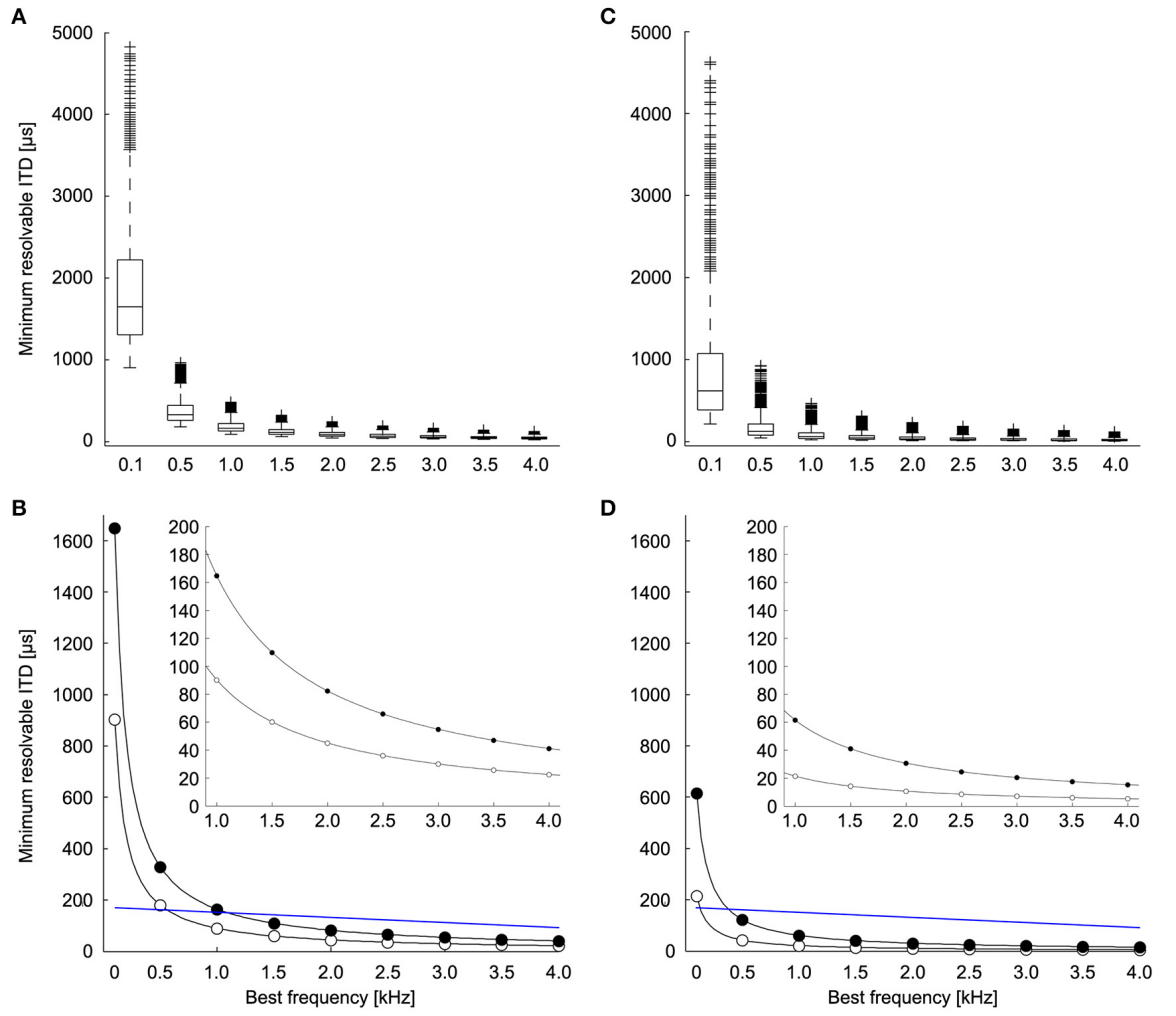
**Minimum resolvable ITD**. **(A)** Minimum resolvable ITD as a function of best frequency. Each boxplot shows the distribution of minimum resolvable ITD if the peak is used to discriminate between ITDs over all combinations of the parameters **(A,B)**, and k (*n* = 1123). The box extends from the first quartile to the third quartile of the sample. Outliers (+) are datapoints that lie >1.5-fold the interquartile range of the sample beyond the box. Lines extend to the maximum and minimum points that are not outliers. **(B)** The minimum (white) and median (black) of the minimum resolvable ITD at each frequency if the peak is used to discriminate between ITDs. Blue line indicates natural range of ITDs (See Methods and Hyson et al., 1994). The inset shows the minimum and median for frequencies between 1 and 4 kHz. **(C)** Minimum resolvable ITD as a function of best frequency if the slope is used to discriminate between ITDs (*n* = 1220). **(D)** The minimum (white) and median (black) of the minimum resolvable ITD at each frequency if the slope is used to discriminate between ITDs. Blue line indicates natural range of ITDs (See Methods and Hyson et al., 1994). The inset shows the minimum and median for frequencies between 1 and 4 kHz.

We calculated the minimum resolvable ITD based on the discrimination of ITDs at both the peaks and the slopes of the ITD tuning curve (Figures 2C,D; adapted from Hyson, 2005). The theoretically possible ITD resolution based on peak discrimination was found to be less than 20% of the phase for most neurons (median = 16.5%, first quartile = 13.0%, third quartile = 22.8%, *n* = 1123). ITD resolution based on the peak of the ITD tuning curve is highly dependent on stimulus frequency (Figure 4A) and becomes smaller with higher frequencies. Given the relationship between Δ*IPD* and Δ*ITD*, the minimum resolvable ITD decreased with frequency as 1/*f*, as expected. There was a range of minimum resolvable ITD values at each frequency because the noise in the model depends on the spike count of the neuron, and thus depends on the parameters for the background firing rate, the dynamic range, and the exponent of the proportional noise. Figure 4B shows the minimum (white dots) and the median (black dots) values for the minimum resolvable ITDs based on peak discrimination. With a natural range of 170 μs at 800 Hz (Hyson et al., 1994) (Or 300 μs for adult chickens; Köppl and Carr, 2008), it becomes infeasible to distinguish ITDs throughout the natural range, based on the peak of the ITD tuning curve at frequencies below 500 Hz.

Considering the limitations of a peak-based ITD coding, it has been suggested that ITD detection in the chicken is based on the slope of the tuning curve rather than the peak (Joseph and Hyson, 1993; McAlpine et al., 2001; Bala et al., 2003; Hyson, 2005) (Figure 2D, adapted from Hyson, 2005). We evaluated ITD resolution over all possible reference ITDs to determine the minimum resolvable ITD. We found that ITD discrimination was highest for reference ITD values slightly below the ITD at the maximum slope (25% of the period) (median = 32.4%, first quartile = 28.4%, third quartile = 63.5%, *n* = 1220). This places the point of best ITD discrimination nearer to the trough of the tuning curve than to the peak. There was a range of minimum resolvable IPDs due to the diversity of neural tuning parameters. For most neurons, Δ*IPD* was found to be less than 10% of the period (median = 6.2%, first quartile = 3.9%, third quartile = 11.0%, *n* = 1220). The minimum resolvable ITD based on the most sensitive part of the ITD tuning curve decreased with frequency as well (Figures 4C,D). Here, the minimum resolvable ITD was significantly better than the peak-based discrimination (*p* < 10^−3^; Mann–Whitney *U*-test) and was as low as 20 μs for some neurons at 1 kHz (Figure 4D, inset). This is approximately four times better than the ITD resolution achieved when the peak of the ITD tuning curve is used (Figure 4B, inset). For all frequencies, but particularly for low frequencies below 2 kHz, ITD resolution is better if the slope of the ITD response is used.

**Figure 5:**
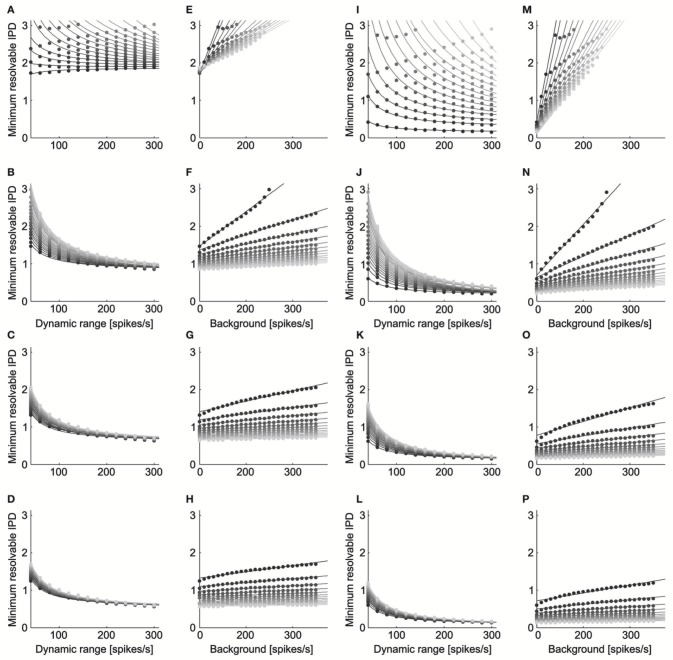
**ITD discrimination depends on dynamic range, background rate, and noise**. The minimum resolvable IPD varied inversely with the dynamic range ($\hat{\Delta IPD}$ = *c*_0_ + *c*_1_/*A*) for each level of background firing rate and noise exponent (Panels **A–D**, peak; Panels **I–L**, slope). The dynamic range is twice the amplitude *A*. The grayscale in **(A–D)** and **(I–L)** codes for the background firing rate, where lighter gray corresponds to higher firing. The noise exponent is constant in each row with *k* = 1 for the top row, *k* = 2 for the second row, *k* = 3 for the third row, and *k* = 4 for the bottom row. The minimum resolvable IPD increased linearly with the background firing rate ($\hat{\Delta IPD}$ = *c*_0_ + *c*_1_ *B*) at each fixed value of the dynamic range and noise exponent (Panels **E–H**, peak; Panels **M–P**, slope). The grayscale in **(E–H)** and **(M–P)** codes for the dynamic range, where lighter gray corresponds to a larger dynamic range. The minimum resolvable IPD was largest when the firing rate noise was proportional to the mean rate (Panels **A,E,I,M**) and decreased as the firing rate noise decreased.

ITD discrimination by the model neurons was best for parameters that led to a high dynamic range and low noise (Figure 5). We found qualitatively similar parameter dependence for ITD discrimination at the peak (Figures 5A–H) and slope (Figures 5I–P) of the ITD curve. The minimum resolvable IPD varied inversely with the dynamic range for each level of background firing rate and noise exponent (Figures 5A,B, peak: mean *r*^2^ = 0.98, *SD* = 0.05, *n* = 93, *p* < 0.004 for each; Figures 5I–L slope: mean *r*^2^ = 0.99, *SD* = 0.008, *n* = 93, *p* < 0.016 for each). This is expected, as IPD discrimination should improve as the difference in the rates produced at different IPDs increases. We also found that the minimum resolvable IPD increased linearly with the background firing rate at each fixed value of the dynamic range and noise exponent (Figures 5E–H, peak: mean *r*^2^ = 0.97, *SD* = 0.01, *n* = 56, *p* < 0.011 for each; Figures 5M–P slope: mean *r*^2^ = 0.96, *SD* = 0.04, *n* = 56, *p* < 0.041 for each). While the background firing rate does not influence the difference between firing rates at different IPDs, increasing the background rate increases the overall firing rate of the neuron and thus increases noise, since the noise increases with the mean rate. As expected, the minimum resolvable IPD was largest when the firing rate noise was proportional to the mean rate (Figures 5A,E,I,M) and decreased as the firing rate noise decreased.

### Allowable best ITDs

**Figure 6:**
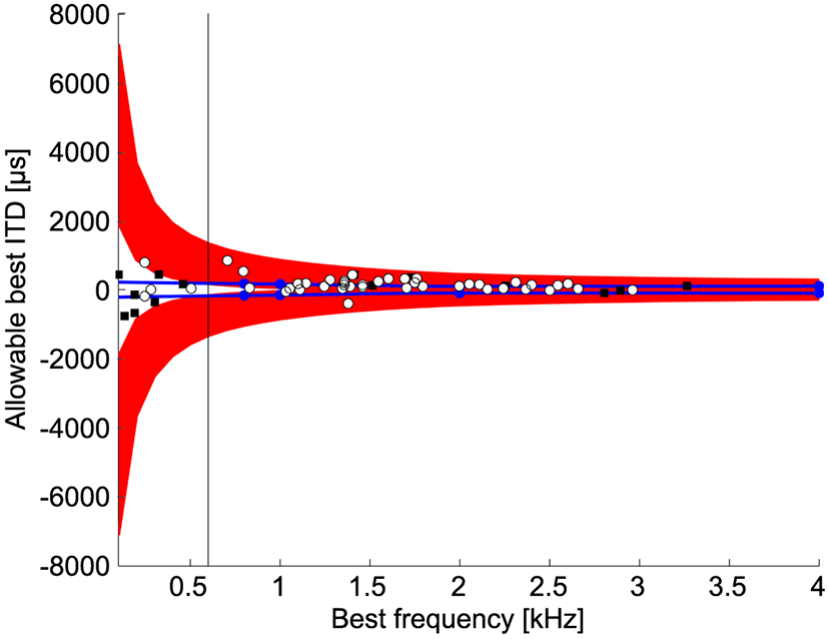
**Allowable best ITDs**. Allowable best ITDs are those where it is possible to detect a difference between two ITDs using the slope of the ITD tuning curve within the natural range. The red regions show the allowable best ITDs. The blue lines indicate the maximum natural ITD of the chicken (See Methods). Blue dots indicate measurements by Hyson et al. (1994). White dots and black squares show the best ITDs of neurophonic and single unit responses, respectively, in chicken NL (Köppl and Carr, 2008). For frequencies below 600 Hz 92% (11/12) of recorded best ITDs are outside the range of allowable best ITDs.

If the slope is used to discriminate between naturally occurring ITDs, then there is a limited range of values for the best ITD so that the slope is contained in the natural range of ITDs. The range of best ITDs of neurons where detection of ITD can occur within the natural range depends on best frequency (Figure 6). For best frequencies less than 600 Hz, the allowable best ITDs were outside the normal range of ITDs (Figure 6). This occurs because the slope covers a large range of ITDs at low best frequencies. Thus, the best ITD must be outside the normal range of ITDs to place the slope within the physiological range of ITDs (Harper and McAlpine, 2004). For best frequencies less than 1 kHz, the range of natural ITDs contained non-allowable best ITDs, i.e., best ITDs that cannot be used for ITD discrimination.

## Discussion

In this study we determined the minimum resolvable ITD of single neurons in the avian NL derived with a computational model that was based on previously published ITD tuning curves (Christianson and Peña, 2006; Köppl and Carr, 2008). We evaluated the ability of single NL neurons to resolve ITDs based on a peak-based mechanism, and when the slope of the ITD function is used to discriminate ITDs. Our results predict that for low frequencies (<~ 500 Hz), the peak of individual ITD tuning curves cannot be used to discriminate ITDs throughout the natural range (Figure 4B). If the maximum slope of the ITD tuning curve is used, a much better ITD resolution can be achieved, predominantly at low frequencies (Figure 4). However, at low frequencies (<~ 600 Hz) best ITDs of NL neurons must be outside the natural range of ITD in order for single neurons to discriminate naturally occurring ITDs.

Jeffress' seminal paper provided an elegant explanation for how ITDs are encoded in the brain (Jeffress, 1948). Anatomically, this model, albeit in a modified version, is embodied by the circuitry in the bird auditory brainstem (Young and Rubel, 1983; Carr and Konishi, 1990; Overholt et al., 1992). Based on *in vitro* experiments where ITDs were simulated, it was however suggested that the maximum response of NL neurons might not be able to provide sufficient resolution for ITD coding in the chicken (Joseph and Hyson, 1993; Hyson, 2005). Instead it was proposed that the slopes of the ITD functions are used to discriminate between ITDs (Figures 2C,D). The concept to use the slope of the ITD function for sound localization was proposed previously (e.g., Rose et al., 1966; Goldberg and Brown, 1969). Most recently, a similar concept was used to interpret ITD coding in the mammalian MSO (McAlpine et al., 2001; Brand et al., 2002). It is critical to address ITD coding using a faithful description of both tuning curve shape and variability, as the solution to whether peak or slope coding is optimal depends on both features of the response (Butts and Goldman, 2006). Here we provide the first evaluation of ITD discrimination by NL neurons using a model that describes the diversity of tuning curves shapes and spiking variability observed in NL.

Our data show that the peaks of the ITD functions of chickens can indeed be used for ITD discrimination for frequencies above 500 Hz. Best ITD responses near zero are however found in chicken NL at frequencies less than 500 Hz (Köppl and Carr, 2008). Our model would predict that these neurons are not useful for sound localization as isolated units. As suggested by previous studies of ITD discrimination (Rose et al., 1966; Goldberg and Brown, 1969; McAlpine et al., 2001; Brand et al., 2002; Takahashi et al., 2003; Köppl and Carr, 2008), we found that the maximum slope of the ITD tuning curve allows the system to resolve much smaller ITDs compared to a peak based discrimination (Figure 4). If the slope of the ITD function is to be used effectively, the best ITD of a low frequency cell must occur outside the physiological range (Figure 6). *In vivo* recordings from NL cells in chickens show that a substantial number of cells have their best ITD outside the physiological range, yet within our calculated range of allowable best ITDs (Figures 5A, 6A in Köppl and Carr, 2008). The broad distribution of allowable best ITDs we find at each frequency is consistent with a modified Jeffress model. The model does not predict a two-channel code at all frequencies, where best IPDs concentrate around a uniform value (McAlpine et al., 2001).

The minimum resolvable ITD from single neuron responses theoretically may be reduced by pooling over NL neurons to reduce variability (Hall, 1965; Yin and Chan, 1988). There is evidence for variability reduction within the frequency channels of the owl's ITD pathway (Christianson and Peña, 2006; Fischer and Konishi, 2008). Based on previously published data (Seidl et al., 2010), the number of neurons in chicken NL of a given isofrequency plane can be estimated: In the low frequency, caudolateral region of NL, a given isofrequency plane consists of 33 neurons, in the high frequency, rostromedial region of NL, 37 NL neurons span across an isofrequency plane (See also Figure 2 in Wang and Rubel, 2008). The small number of neurons per isofrequency plane in the chicken suggests that there is a limited opportunity for pooling to reduce the minimum resolvable ITD in downstream neurons. Therefore, the minimum resolvable ITD found in NL in the chicken places limitations on the frequencies that can be useful for sound localization behavior. Pooling of neurons may however be a secondary phenomenon: In mammals, single neurons are able to encode behaviorally relevant ITDs (Skottun, 1998; Skottun et al., 2001). It remains an open question how pooling may occur for NL neurons at the lowest frequencies where naturally occurring ITDs cannot be distinguished from neural responses. As proposed by Köppl and Carr (2008), and supported by our analysis, it is possible that these neurons are used for decorrelation detection and not sound localization. Alternatively, ITD discrimination may be based on a population of neurons, which may improve ITD resolution beyond that provided by individual neurons (Fitzpatrick et al., 1997).

The code for ITD in the avian auditory system has been addressed in previous studies (Harper and McAlpine, 2004; Goodman et al., 2013; Goeckel et al., 2014). Harper and McAlpine (2004) investigated the optimal code for ITD as a function of frequency in avian and mammalian systems. They found the distribution of best ITDs at each frequency that maximized the population Fisher information. Through the maximization of the population Fisher information, they conclude that the optimal representation is a slope code at low frequencies and a place code at high frequencies. The cutoff between high and low frequencies varies with the head size of the animal. Harper and McAlpine (2004) addressed the issue of ITD coding in a similar, but in a complementary way to our analysis. The use of the population Fisher information as the objective function means that their analysis is not computing a direct measure of how well individual neurons can resolve ITD. Moreover, focusing on the representation of ITD that maximizes the Fisher information, rather than addressing the single neuron Fisher information, means that a representation may be the best possible at a frequency, yet still not accurate enough to allow for discrimination of ITDs within the natural range. Here we determine how well single neurons discriminate ITD and whether this performance is ethologically significant. Our work uses the ROC analysis to precisely quantify the limits of ITD discrimination by avian NL neurons. This differs from Harper and McAlpine's use of the Fisher information to provide a bound on the discrimination performance. Our conclusions confirm Harper and McAlpine's result that a peak code is not possible at the lowest frequencies (Harper and McAlpine, 2004). We extend their work by showing the specific discrimination performance of NL neurons and determining the range of best ITDs that will allow for ITD discrimination within the natural range.

Our analysis places constraints on the code for ITD in the avian auditory system, but does not address the form of the optimal population code. To determine the optimal population code for ITD, characteristic delays and phases must be considered (Lüling et al., 2011; Goodman et al., 2013). Characteristic phases were not explicitly included in our model, as we only considered the best phase. The optimal population code depends not only on the information content in neural responses, but the mechanism used to decode the responses (Fischer and Peña, 2011). The form of the population code may be reshaped throughout the auditory pathway depending on task demands and decoding methods (Bala et al., 2003, 2007; Vonderschen and Wagner, 2014).

Other studies have addressed the question of whether single neurons or a population of neurons are able to encode ITDs (Fitzpatrick et al., 1997; Skottun, 1998; Skottun et al., 2001; Shackleton et al., 2003, 2005). Our results are consistent with previous studies in mammals which showed that ITD discrimination improves with frequency and is best near the point of steepest slope (Skottun, 1998; Skottun et al., 2001; Shackleton et al., 2003). The resolution of ITD by single cells in previous studies is sufficient to reflect behavioral data (Skottun, 1998; Skottun et al., 2001; Shackleton et al., 2003). The ITD discrimination of some model NL neurons at frequencies above 3 kHz matches the ITD discrimination performance of the owl (Bala et al., 2003), however it is unlikely that only one neuron is involved in a specific sound localization task.

Köppl and Carr (2008) describe ITD sensitive NL neurons with best frequencies as low as 80 Hz. Evidence for a delay line structure in low frequency regions of the barn owl NL is inconclusive (Köppl and Carr, 1997) and physiological data does not necessarily support a place code for lower frequencies (Wagner et al., 2007). The barn owl however appears to be a specialized animal amongst birds (Kubke and Carr, 2000; Ashida and Carr, 2011), as other avian species display a distinct single cell layer in NL (e.g., chicken: Jhaveri and Morest, 1982; Wang and Rubel, 2008; quail: Seidl et al., 2013; emu: MacLeod et al., 2006). It is unknown to what degree chickens need to resolve ITDs at lower frequencies. But it seems unlikely that a sophisticated mechanism like ITD processing is preserved in an animal when it loses its function.

The frequency-specific ITD resolution may reflect an adaption to communication calls of chickens. Calls of newly hatched chicks are found to be above 2 kHz and thus in a range in which we determined minimum resolvable ITDs based on peak discrimination to be in the natural range (Figure 4) (Wood-Gush, 1971). Most adult communication calls contain a high frequency component, hence behaviorally relevant sound localization behavior appears to be possible with the values we determined (Wood-Gush, 1971).

Our results have implications for the time window in which binaural excitatory inputs have to coincide at individual NL neurons. According to our model, the theoretical resolution of ITDs can be as low as 10 μs for some frequencies (Figure 4). This would require binaural excitatory inputs to arrive within a small microsecond time window. Conduction time along NM axons is regulated in a temporally precise manner by systematic variations of axon diameter and internode distance (Seidl et al., 2010, 2014). In other words, signal propagation time of an action potential along NM axons that are 1480 and 3166 μm long (Seidl et al., 2010) must be adjusted accurately. The temporal rigor of this conduction velocity regulation suggests an activity-dependent neuron-glia interaction responsible for these variations (Rasband, 2010; Seidl, 2013).

This study determined theoretically resolvable ITDs in the chicken NL based on a computational model. Our simulation predicts that peak-based ITD coding is not useful at low frequencies in the way classically predicted by the Jeffress model. That is, the ITD resolution of single neurons based on responses near the peak is too low to be useful at low frequencies. The maximum slope of the ITD function can be used to achieve a much higher ITD resolution at all frequencies compared to a peak-based ITD discrimination. Together with others (Takahashi et al., 2003; Köppl and Carr, 2008) we propose that the slope of the ITD response in the chicken NL may be used for other tasks than sound localization, such as decorrelation detection. Moreover, our results provide an important reference for studies evaluating the conduction time development and regulation along NM axons, as the range of resolvable ITDs dictates the time window in which binaural excitation must occur to elicit an action potential in NL.

## Materials and methods

### ROC analysis

We used ROC analysis to calculate the minimum resolvable IPD for model neurons (Bradley et al., 1987; Skottun, 1998; Skottun et al., 2001; Köppl and Carr, 2008). For each IPD curve we used a range of reference and test IPDs and computed the percent correct in discriminating the test IPD from the reference IPD based on noisy responses of the model NL neuron. For a given reference IPD, the minimum resolvable IPD was the smallest distance to a test IPD that yielded 75% correct discrimination performance. The minimum resolvable IPD for the neuron was the smallest value obtained over all reference IPDs. The reference IPD where the minimum resolvable IPD occurred was designated as the most sensitive IPD. If the percent correct discrimination performance did not reach 75% for any IPD, then we did not include a minimum resolvable IPD for that model neuron.

ROC analysis was used to determine the percent correct in the task of discriminating between two IPDs based on the firing rate of the model neuron. This analysis uses the probability distributions of the responses at the test and reference IPDs, which in our model are Gaussians. To decide which IPD produced a given firing rate, the rate is compared to a threshold. If the firing rate is above the threshold, the decision is that the stimulus was the reference IPD. Conversely, if the firing rate is below the threshold, then the decision is that the stimulus was the test IPD. The difficulty of the task depends on the overlap of the firing rate distributions at the two IPDs and is characterized by two probabilities: the hit rate and the false alarm rate. The false alarm rate is the probability of the rate being above threshold when the IPD is the test IPD. The hit rate is the probability that the rate is above threshold when the IPD is the reference IPD. The ROC curve plots the hit rate against the false alarm rate for different values of the threshold. The area under the ROC curve is equal to the percent correct in the decision task. The area under the ROC curve will be one when the firing rate distributions for the test and reference IPDs do not overlap at all. At the other extreme, the area under the curve will be 0.5 if the distributions overlap completely and performance is chance.

### Natural range of ITDs in the chicken

We estimated the natural range of ITD as a function of frequency from the data of Hyson et al. (1994; Köppl and Carr, 2008). The natural range of ITDs in the chicken may rely on internal acoustical coupling (Calford and Piddington, 1988). We used the Matlab function grabit.m (MathWorks, Natick, MA) to determine the measured ITD as a function of frequency from Figure 3 in Hyson et al. (1994). The maximum ITD at each frequency was taken as the average of the maxima in the positive and negative directions. We used cubic Hermite interpolation to determine the maximum ITD at frequencies between 0.8 and 4 kHz, and extrapolation at frequencies below 0.8 kHz where measurements were unavailable. The values obtained from Figure 3 in Hyson et al. (1994) are 169.62, 158.23, 96.2, and 102.53 μs, for 0.8, 1, 2, and 4 kHz, respectively.

### Conflict of interest statement

The authors declare that the research was conducted in the absence of any commercial or financial relationships that could be construed as a potential conflict of interest.
